# Exploring Community Pharmacists’ Awareness, Attitudes, and Experiences with Digital Health Technologies: A Focus on Mobile Applications for Diabetes Mellitus Self-Management

**DOI:** 10.3390/pharmacy14020039

**Published:** 2026-03-02

**Authors:** Dušan Vukmirović, Dušanka Krajnović, Marina Odalović

**Affiliations:** 1 Faculty of Pharmacy, University of Belgrade, Vojvode Stepe 450, 11221 Belgrade, Serbia; 2 Department of Social Pharmacy and Pharmaceutical Legislation, Faculty of Pharmacy, University of Belgrade, Vojvode Stepe 450, 11221 Belgrade, Serbia; dusica.krajnovic@pharmacy.bg.ac.rs (D.K.); marina.odalovic@pharmacy.bg.ac.rs (M.O.)

**Keywords:** digital health technology literacy, mobile health applications, continuing professional development, chronic disease management, pharmaceutical care

## Abstract

Diabetes mellitus is a growing global health challenge, and digital health technologies offer new opportunities to support self-management. Mobile applications can benefit both patients and healthcare professionals; however, awareness and integration of these tools into community pharmacy practice remain limited. As accessible frontline providers, pharmacists are well positioned to promote digital health, yet their readiness and engagement require further investigation. A cross-sectional survey was conducted among community pharmacists in Serbia using a structured questionnaire. Developed through a consensus-based process, the instrument assessed pharmacists’ awareness, attitudes, and experiences with digital health technologies, focusing on mobile applications for diabetes self-management. Only 15.8% of pharmacists were aware of such applications, and 2.4% reported receiving relevant training. Higher digital health technology literacy was associated with greater awareness, confidence, and preference for digital learning. Most participants supported expanding pharmacists’ roles in advising patients on digital tools and expressed interest in structured education and official guidance. These findings indicate limited awareness and training in mobile health applications among community pharmacists. Enhancing digital competencies through targeted education and structured guidance may facilitate greater integration of digital tools into routine pharmacy practice and strengthen pharmacists’ roles in chronic disease management.

## 1. Introduction

Chronic non-communicable diseases (NCDs) remain the leading cause of mortality and morbidity globally. Among them, diabetes mellitus (DM) is a major public health concern, affecting over 500 million people worldwide, and contributing to more than 2 million deaths annually [1]. Its rising incidence has led many to label it a modern preventable pandemic due to its widespread and persistent nature [2].

The rapid advancement of digital technologies in recent years has opened new avenues for the prevention, monitoring, and management of NCDs. One notable area of growth is the development of mobile and software applications designed specifically to support self-management of diabetes by patients and aid healthcare professionals (HCPs) in clinical decision-making [3].

As pharmacy practice evolves, pharmacists are increasingly expected to become “digital pharmacists”, capable of leveraging digital tools and technologies to enhance patient care. This transformation requires expanded digital literacy, updated education, and infrastructural support to fully realize the potential benefits of digital health [4,5]. This perspective aligns with the International Pharmaceutical Federation (FIP), which identifies digital health competence as a core requirement for the future pharmaceutical workforce and emphasizes the need for systematic integration of digital technologies into pharmacy education and practice [6,7].

In Serbia, continuing professional development (CPD) is a legal requirement for pharmacists employed in the healthcare sector (community and hospital pharmacies), regulated by national legislation and overseen by the Pharmaceutical Chamber of Serbia. The National Competency Framework for community pharmacists provides a professional reference for competency development and recognizes the use of information technologies and digital tools in pharmacy practice [8]. While digital health competencies are not explicitly mandated within CPD, elements of healthcare digitalization, such as electronic prescribing, electronic health records, and pharmacy information systems, are included in selected training programs. Structured training specifically focused on broader digital health solutions remains limited.

A consensus report by the European Association for the Study of Diabetes (EASD) and the American Diabetes Association (ADA) classifies diabetes-related digital health applications into three broad categories: those designed for general health and wellness monitoring, those functioning as standalone medical devices, and those that interface with data from medical devices to support diabetes care [9].

These tools offer potential benefits in improving glycemic control, medication adherence, and lifestyle modification. The report also emphasizes that HCPs should be aware of both the benefits and limitations of such technologies and actively guide patients in their proper use [9]. Recent research reinforces this call, noting that as digital tools become more sophisticated and widely adopted, HCPs require clearer guidelines and training to effectively incorporate them into routine practice [10]. This is particularly important in community settings, where frontline providers can play a key role in bridging the digital divide. Global professional bodies, including FIP, similarly stress the responsibility of pharmacists to support patients in navigating digital health tools in a safe, effective, and patient-centered manner [11].

Community pharmacists, due to their high accessibility and frequent patient contact, are strategically positioned to promote digital health literacy and support the use of digital tools for diabetes care. Studies show that pharmacists recognize the potential of digital platforms, such as electronic health records, teleconsultations, and mobile applications, to enhance patient engagement and outcomes, especially in underserved areas [10,12]. However, they also face challenges, including inadequate digital infrastructure, data privacy concerns, and a lack of specialized training [12,13,14].

While pharmacist-led digital interventions have shown promise in improving diabetes outcomes, few studies have explored community pharmacists’ awareness, attitudes, and practical experiences with mobile applications tailored for diabetes self-management. A recent systematic review of healthcare professionals’ perceptions, including those of pharmacists, on marketed mobile applications for type 2 diabetes self-management, highlighted generally positive attitudes but also identified significant gaps in awareness and confidence, particularly among community pharmacists [15]. For instance, one of the studies included found that only 56% of community pharmacists were aware of health apps, and of those, 60% recommended them to patients.

Moreover, a separate systematic review of pharmacist-led digital health interventions, including mobile applications, confirmed measurable benefits in glycemic control and medication adherence, underlining the clinical value of these tools when effectively implemented [16].

Although much of the existing literature originates from high-income countries with well-established digital infrastructures, Serbia, like many low and middle-income nations, offers a valuable lens through which to explore both the opportunities and challenges faced by community pharmacists in engaging with digital health tools. Insights from this context not only address a national evidence gap but also contribute transferable lessons for other countries navigating similar digital transitions in primary care settings.

The aim of this study is to evaluate the awareness, attitudes, and experiences of community pharmacists in Serbia concerning digital health technologies, with a particular emphasis on the use of mobile applications for the self-management of DM.

## 2. Materials and Methods

The study employed a structured questionnaire developed following an extensive literature review and preliminary exploratory work [15,17].

The first section collected sociodemographic and professional characteristics of the participants (7 items). The second section included the Serbian version of the Digital Health Technology Literacy–Assessment Questionnaire (DHTL-AQ), comprising 5 items, which has previously undergone full psychometric validation for use among pharmacists [18]. In the present study, the DHTL-AQ was used to stratify respondents according to their level of digital health technology literacy. Analyses of DHTL group characteristics and cut-off value determination are reported in a separate publication [19], while the present study focuses on pharmacists’ awareness, attitudes, and experiences with digital health technologies and diabetes-related mobile application use.

The third section, entitled “Mobile Applications for Patients with Diabetes,” comprised 26 author-developed items and represented the primary focus of the present study. These items were generated based on a targeted literature review and refined through expert review and an iterative consensus process among the authors to ensure content relevance, clarity, and suitability for the target population. This section was designed to assess community pharmacists’ awareness, attitudes, and experiences related to digital health technologies, with a particular emphasis on mobile applications for diabetes mellitus self-management.

### 2.1. Study Design and Setting

This study employed a cross-sectional design using both online and paper-based questionnaires to collect data from community pharmacists in Serbia. The entire population of community pharmacists was informed about the study and its objectives and invited to participate through an email notification facilitated by The Pharmaceutical Chamber of Serbia. The email included a link that provided access to the online version of the questionnaire.

To complement online data collection, identical paper questionnaires were distributed in parallel via direct fieldwork across community pharmacies. A representative sample was achieved by offering the paper-based survey to pharmacists working in both major pharmacy chains and independent pharmacies, spanning diverse geographic locations-from urban centers to rural areas across multiple regions of the country.

Data collection began on 19 December 2023 and continued until the targeted sample size was reached on 15 May 2024.

### 2.2. Participants and Data Collection

This study focused on community pharmacists employed in community pharmacies who voluntarily consented to participate without incentives and agreed to the use of their responses for scientific purposes. The selection process aimed to ensure broad representation across various pharmacy settings, aligning with the study’s objective.

To determine the necessary sample size, calculations were based on the total population of community pharmacists in Serbia, as verified by the Pharmaceutical Chamber of Serbia (7207 pharmacists). A representative sample of at least 365 participants was required, following a standard calculation with a 95% confidence level and a 5% margin of error [20].

Data collection combined both digital and in-person methods. Online responses were gathered via Google Forms, ensuring accessibility for participants across different regions. Paper-based surveys were completed privately and retrieved at designated collection points within pharmacies, supporting engagement from pharmacists in various practice environments. To prevent duplicate participation, respondents were asked whether they had previously completed the questionnaire. In the digital version, participants who indicated they had already completed the survey were automatically redirected to the end of the questionnaire with a thank-you message. All submitted responses remained anonymous, with access restricted solely to the research team.

### 2.3. Data Analysis

Statistical analysis was conducted using IBM SPSS Statistics for Windows, version 26 (IBM Corp., Armonk, NY, USA). Descriptive statistics were applied to summarize sample characteristics: categorical variables (gender, education level, pharmacy type, pharmacy location, diabetes consultancy, perception of digital health, eHealth, and mHealth) were presented as frequencies, while continuous variables (duration of working experience) were described using means, standard deviations, medians, and interquartile ranges.

To assess statistical significance in the difference between categories, the chi-square (χ^2^) test was employed for categorical variables, and the Kruskal–Wallis test was used for comparisons involving nonparametric data between groups.

## 3. Results

This study explored community pharmacists’ awareness, attitudes, and experiences with digital health technologies, with a particular focus on mobile applications for diabetes mellitus self-management. The study sample comprised 368 community pharmacists from various regions of Serbia. Participants had a mean professional experience of 13.9 ± 9.8 years. The majority were female (89.1%), held a Master of Pharmacy degree (83.4%), and were employed in private pharmacy chains (76.4%). With respect to geographic distribution, most respondents worked in large urban areas with more than 150,000 inhabitants (63.3%), followed by medium-sized cities (19.3%), small towns (11.4%), and rural settings (6.0%). Additionally, 8.4% of participants reported working as certified diabetes consultants.

Further details regarding participants’ sociodemographic and professional characteristics according to awareness of different categories of mobile applications are summarized in Table 1.

The majority of community pharmacists (Table 2) reported a lack of awareness with mobile applications designed to support a healthy lifestyle (77.2%) and those intended for disease self-management and therapy management (84.2%) (Supplementary Materials Table S1).

Results for mobile applications promoting a healthy lifestyle that were more widely recognized among pharmacists demonstrated a statistically significant difference (χ^2^(2) = 8.40, *p* = 0.02) across different levels of DHTL, indicating that pharmacists with higher DHTL scores were more likely to be aware of such applications. Post hoc analysis revealed that participants with high DHTL were significantly more likely than expected to report awareness (Z = +2.9, *p* < 0.01), while those with low DHTL were significantly more likely to report unawareness (Z = +2.1, *p* < 0.05). Conversely, high DHTL participants were significantly less likely to report unawareness (Z = −2.9, *p* < 0.01).

When asked how they became aware of mobile applications supporting a healthy lifestyle, most respondents reported internet searches (13.3%) or discussions with colleagues and other health professionals (4.9%), while a smaller proportion cited professional literature (1.4%). The most common response overall, however, was a lack of awareness (77.2%), which was particularly pronounced among respondents with low DHTL levels (83.3%; 110 out of 132), compared to medium (81.9%; 59 out of 72) and high (70.1%; 115 out of 164) DHTL groups (Fisher’s Exact Test, *p* = 0.008).

The method of discovering mobile health applications varied significantly by DHTL level. Internet search was the most frequently cited source among those aware of such applications, particularly among high DHTL participants (20.1%; 33 out of 164), compared to medium (8.3%; 6 out of 72) and low (7.6%; 10 out of 132) groups. Post hoc analysis revealed that high DHTL participants were significantly more likely to report internet searches as their source of awareness (Z = +3.4, *p* < 0.01), whereas low DHTL participants were significantly more likely than expected to report no awareness (Z = +2.1, *p* < 0.05). Conversely, high DHTL participants were significantly less likely to report being unaware (Z = −2.9, *p* < 0.01). No significant group differences were observed for other information sources (all |Z| < 1.96).

When it comes to the developments in the field of diabetes, pharmacists reported that they stay informed primarily through CPD courses (92.93%) and conversations with colleagues and other healthcare professionals (79.35%). Other significant sources include pharmaceutical companies (63.32%), professional journals and literature (54.62%), and talking to patients (53.53%). Internet searches are used less frequently (41.30%), with no participants indicating that they do not keep up to date with diabetes news.

Among pharmacists who use internet searches to stay informed about diabetes, the most frequently accessed website category was unspecified (25.00%). This was followed by general medical education portals (22.37%) and websites of diabetes-related organizations (20.39%). Pharmacists also commonly accessed websites of pharmacists’ organizations (19.74%) and regulatory or health institutions (19.74%). Less frequently used sources included medical literature databases (16.45%), drug information databases (9.21%), and search engines (5.26%). Websites of healthcare product manufacturers or wholesalers were accessed by 3.29% of pharmacists, while social media platforms were the least utilized source (0.66%).

Table 3 presents the different categories of mobile applications mentioned by community pharmacists, covering both those supporting a healthy lifestyle and those focused on diabetes self-management and therapy management.

The most frequently mentioned applications are Samsung Health, Apple Health, and HUAWEI Health; they all belong to the category for monitoring general health and well-being, and they were mentioned by 30.95% of pharmacists who indicated that they were aware of the existence of this type of application.

Pharmacists generally believe that patients with diabetes are interested in using mobile applications for disease management (89.4%). However, the majority perceive this interest as partial (43.5%) or rare (36.1%).

Only a small proportion of pharmacists (4.9–5.2%) provided feedback on the support they provided as community pharmacists to patients regarding mobile applications for diabetes self-management. Among those who provided feedback, more than 50% fell into the high DHTL category. The areas of support provided to both patients who already use and those who do not use mobile applications are detailed in Table 4.

Overall, a limited number of pharmacists reported receiving training on digital technologies. Specifically, only 2.4% had undergone training related to mobile applications for diabetes self-management, while 11.7% reported receiving training on other digital technologies. Detailed information is provided in Table 5.

A majority of pharmacists (58.4%) indicated that knowledge of mobile applications for diabetes self-management would be helpful either “almost always” (14.9%) or “often” (43.5%). Additionally, 29.3% believed such knowledge was partially helpful, while 7.3% and 4.9% considered it “rarely” or “not at all” helpful, respectively. A linear-by-linear association test showed a significant trend between DHTL level and perceived usefulness of mobile application knowledge, with higher DHTL scores associated with greater perceived usefulness (χ^2^(1) = 15.04, *p* < 0.001). Post hoc adjusted residuals indicated that pharmacists with high DHTL scores were more likely to perceive such knowledge as “almost always” helpful (Z = +2.8, *p* < 0.01), whereas those with low DHTL scores were more likely to perceive it as “not at all” (Z = +2.3, *p* < 0.05) or “rarely” helpful (Z = +1.8, *p* = 0.07).

A significant proportion of pharmacists believe they should provide guidance and training to patients on the appropriate use of mobile applications designed to support a healthy lifestyle (70.7%), as well as those facilitating disease self-management and therapy management (78.3%).

Additionally, pharmacists recognize the need for further training, both in general digital technologies applicable to pharmaceutical healthcare (87.5%) and in specific mobile applications tailored to support patients with diabetes (89.7%).

Face-to-face education was the most commonly selected method overall (42.1%), and was significantly more preferred by respondents with low DHTL (49.7%) than by those with medium (16.1%) or high DHTL (34.2%) (χ^2^(4) = 23.08, *p* < 0.001). Post hoc analysis confirmed this pattern, with low DHTL participants more likely than expected to choose in-person education (Z = +4.7, *p* < 0.001), and high DHTL participants significantly less likely than expected to prefer this format (Z = −3.4, *p* < 0.001). Conversely, self-paced online education was most popular among high DHTL participants (52.8%), while only 23.9% of low DHTL and 23.2% of medium DHTL participants chose this option. Post hoc residuals supported this, showing high DHTL respondents were significantly more likely to choose this format (Z = +2.5, *p* < 0.01), and low DHTL respondents significantly less likely (Z = −3.8, *p* < 0.001). Although real-time online education was selected more often by those with high DHTL (50.7%) compared to medium (19.7%) and low (29.6%), this difference was not statistically significant based on adjusted residuals (all Z < 1.96).

When asked how they would like to receive updates about digital technologies, the most preferred method overall was via email (52.2%), especially among high DHTL participants (49.0%), compared to medium (22.4%) and low (28.6%) (χ^2^(4) = 10.54, *p* = 0.03). However, post hoc analysis showed only the low DHTL group had a significant deviation, being less likely than expected to prefer email (Z = −3.0, *p* < 0.01). The higher preference among high DHTL respondents was not statistically significant (Z = +1.8). Continuing Professional Development (CPD) courses were selected by 42.4% overall and were significantly more preferred by low DHTL participants (Z = +3.1, *p* < 0.01), indicating a preference for more structured learning formats in this group. Professional publications were the least chosen method (5.4%), and no significant differences across DHTL groups were found (all Z < 1.96).

The vast majority of pharmacists (92.1%) believe that official guidelines on mobile applications for diabetes management would be beneficial in their work with diabetic patients.

## 4. Discussion

A key finding of this study is the limited awareness among community pharmacists of mobile health applications, particularly those designed for disease self-management and therapy support. This aligns with the 2025 FIP report on Pharmacy’s Role in the Digital Transformation of Health, which highlights the critical role of pharmacists in guiding patients through digital tools while noting global variability in workforce readiness [21]. Despite the growing availability of such tools, most respondents were not familiar with their existence. Awareness was higher for applications promoting a healthy lifestyle than for those supporting disease or therapy management, suggesting that wellness-focused apps may be more visible or better integrated into public health messaging. Importantly, awareness was associated with DHTL, indicating that pharmacists with higher DHTL were more likely to recognize these tools. This finding is consistent with our previous report, where the majority of community pharmacies (64.1%) exhibited medium or high levels of DHTL, and significant differences were observed across groups with varying lengths of professional experience [19]. Together, these results suggest that digital competence plays a significant role in whether healthcare professionals are equipped to identify and potentially recommend mobile health technologies to patients. These findings highlight the need for targeted interventions aimed at improving digital literacy and integrating digital health awareness into professional education and continuing development frameworks [22]. This is consistent with FIP Development Goal 20, which calls for the development of a digitally competent pharmacy workforce capable of integrating digital tools into practice [7].

A substantial gap in awareness of mobile applications for promoting a healthy lifestyle among pharmacists was observed, with 77.2% of respondents indicating they were unaware of such tools. Even among pharmacists with higher DHTL, 70.1% still reported being unaware. Internet searches were the most commonly cited source of awareness among those familiar with mobile applications, particularly among high DHTL pharmacists, while those with lower DHTL levels were significantly more likely to report no awareness. These patterns suggest that limited digital navigation skills or reduced confidence in engaging with digital health technologies may contribute to lower discovery rates in less digitally literate groups.

These results are consistent with prior research. For instance, one study reported limited knowledge of mHealth apps among pharmacists and pharmacy students in Saudi Arabia, despite generally positive attitudes toward their use [23]. Similarly, systematic reviews indicate that while mHealth apps can effectively support diabetes adherence and monitoring, their real-world uptake is often constrained by barriers such as lack of awareness, low digital literacy, and limited user engagement [24,25]. The present study reinforces these findings within a new context and highlights the urgency of designing targeted education and awareness-raising interventions.

In addition to the primary findings, insights into how pharmacists stay informed about diabetes developments offer valuable context. Most pharmacists reported relying on Continuing Professional Development (CPD) courses and peer discussions, while internet searches and professional literature were less commonly used. Social media platforms were the least utilized source. General medical education portals and diabetes-specific organizational websites were the most frequently accessed online sources. These patterns suggest a need to optimize dissemination strategies by aligning educational outreach with pharmacists’ preferred information channels.

The majority of pharmacists believe that patients with diabetes are at least somewhat interested in using mobile apps. Many also felt that pharmacists should play a role in supporting and advising patients on the use of these tools. Furthermore, most respondents agreed that knowledge of mobile applications for diabetes self-management is useful in their everyday work, particularly among those with higher DHTL scores. This aligns with other findings showing that pharmacists generally perceive mHealth tools as beneficial in theory, despite limited practical familiarity [23].

However, few pharmacists in this study reported receiving formal training on digital technologies. Only 2.4% indicated having training on mobile apps, and just 11.7% had received training on other digital technologies. This suggests a possible disconnect between pharmacists’ professional development and the evolving digital landscape of healthcare. The low uptake may also reflect low retention, inadequate emphasis, or a lack of visibility of available digital training programs. This gap mirrors observations in the FIP report, which emphasizes the urgent need to equip pharmacists with digital competencies through formal education and continuing professional development [6].

Encouragingly, most respondents recognized the need for further education. Specifically, 87.5% expressed interest in additional training on general digital technologies relevant to pharmaceutical care, and 89.7% supported training on mobile apps for diabetes support. This demand highlights a significant gap between current exposure and perceived need.

Furthermore, a strong majority (92.1%) of participants supported the development of official guidelines for the use of mobile applications in diabetes care. This suggests that pharmacists would likely welcome standardized, evidence-based resources to support their integration of digital tools into everyday practice. The development of such guidelines could be a critical step toward better equipping pharmacists to support patients with chronic conditions like diabetes.

Preferences for educational delivery also varied by digital literacy. Pharmacists with lower DHTL preferred in-person formats and structured continuing education, while those with higher DHTL favored self-paced online modules and email-based updates. These results reflect the influence of digital competence on comfort with independent, technology-mediated learning, consistent with earlier findings in healthcare education [26]. For example, structured approaches are often required to build confidence in digital health instruction among healthcare professionals [27]. Additionally, high interest in digital health has been observed among medical students, though digital readiness varies significantly depending on previous exposure and confidence [28]. Tailoring educational formats to pharmacists’ digital competencies could enhance the uptake and effectiveness of mHealth technologies in practice.

These findings are also in line with broader international trends. A recent study conducted among community pharmacists in the UK found that although many acknowledged the increasing importance of digital health technologies, confidence in their use and recommendation within patient care remained limited [29]. This further highlights the global relevance of structured digital training and practice integration strategies.

This study has several methodological limitations. First, although both online and paper-based surveys were distributed to increase sample diversity, the sampling strategy was not random. This may have introduced selection bias, particularly if participants opting for the online survey were more digitally literate. To mitigate this, alternative formats were offered to accommodate a broader range of digital skill levels. Second, the reliance on self-reported data introduces potential for social desirability bias and recall inaccuracies. Third, the cross-sectional design limits causal interpretations and captures a single point in time, which may not reflect ongoing changes in technology adoption or literacy. Finally, pharmacists’ age was not collected, as years of experience in community pharmacy were prioritized as a more practice-relevant indicator of exposure to digital technologies. Given the conceptual overlap and potential multicollinearity between age and professional experience, only the latter was retained in the analysis. As previously reported in our research [19], significant differences in DHTL levels were associated with years of community pharmacy experience, which further supports this choice. In Serbia, years of community pharmacy experience generally correlate with age, since pharmacists typically enter practice soon after graduation and often remain in this setting long term. Although this relationship is not absolute, excluding age may limit the ability to assess its independent influence on digital literacy and technology acceptance and should be considered in future research.

In addition, data collection spanned a five-month period, which allowed partial coverage of potential seasonal variations in pharmacy workload and patient demand. This extended timeframe may enhance the representativeness of responses by capturing practice conditions across different periods of the year. At the same time, recruitment overlapped with several public and religious holidays in Serbia, which may have influenced pharmacist availability and response rates. These contextual factors may be relevant for future researchers when planning recruitment strategies and timelines.

The observed gaps in awareness and training highlight important opportunities to better equip pharmacists for digital health integration. Tailored interventions should consider pharmacist demographics and digital literacy: for example, modular, time-efficient training, peer mentoring, or continuing education incentives may help engage those with lower digital confidence. FIP’s Statement of Policy on Digital Health recommends structured workforce development, guidance, and supportive infrastructure to enable pharmacists to deliver safe, effective, and patient-centered digital services [11].

In particular, the results suggest missed opportunities to expand pharmacists’ roles in supporting patients with mobile health applications for chronic disease self-management. By embracing digital technologies, pharmacists can develop new patient-centered services that go beyond traditional functions. This evolution is critical to meeting the changing expectations of healthcare systems in the digital era and safeguarding the future relevance of the pharmacy profession.

Future research should evaluate structured education models matched to varying DHTL levels, test standardized search protocols and resource bundles to assist pharmacists in identifying credible digital tools, and explore how digital literacy influences pharmacist–patient communication and the uptake of mHealth in diabetes care. In addition, future studies could examine demographic factors influencing participation in training and investigate barriers among pharmacists who do not perceive training as helpful to better inform targeted educational strategies.

## 5. Conclusions

This study reveals that although many community pharmacists recognize the value of mobile health applications in diabetes care, their awareness and training remain limited, especially regarding disease-specific tools. Awareness and perceived usefulness were positively associated with digital health technology literacy. However, structured training on digital technologies is largely lacking, and pharmacists express a clear need for further education and official guidance. Enhancing digital competence and integrating mHealth tools into routine pharmacy practice will be essential to ensure pharmacists can effectively support patients with chronic conditions like diabetes. While these findings are context-specific and based on cross-sectional data, they highlight critical areas for professional development and future research.

## Figures and Tables

**Table 1:** Sociodemographic and professional characteristics of community pharmacists according to awareness of mobile applications for diabetes care.

Characteristic	Total N (%)	Awareness of Mobile Apps Supporting a Healthy Lifestyle		Awareness of Mobile Apps Supporting Disease Self-Management and Therapy	
		Yes N (%)	No N (%)	Yes N (%)	No N (%)
Gender					
Female	328 (89.1%)	68 (20.7%)	260 (79.3%)	58 (15.8%)	310 (84.2%)
Male	40 (10.9%)	16 (40.0%)	24 (60.0%)	10 (25.0%)	30 (75.0%)
Pharmacy type					
Pharmacies within Pharmacy Chains (state property)	39 (10.6%)	15 (38.5%)	24 (61.5%)	8 (20.5%)	31 (79.5%)
Pharmacies within Pharmacy Chains (private)	281 (76.4%)	63 (22.4%)	218 (77.6%)	43 (15.3%)	238 (84.7%)
Independent pharmacies	48 (13.0%)	6 (12.5%)	42 (87.5%)	7 (14.6%)	41 (85.4%)
Degree of education					
Master of Pharmacy	307 (83.4%)	72 (23.5%)	235 (76.5%)	47 (15.3%)	260 (84.7%)
Master of pharmacy—specialist	58 (15.8%)	12 (20.7%)	46 (79.3%)	11 (19.0%)	47 (81.0%)
Master of Pharmacy-Doctor of Science	3 (0.8%)	0 (0.0%)	3 (100%)	0 (0.0%)	3 (100%)
Pharmacy location					
Large city (>150,000 inhabitants)	233 (63.3%)	60 (25.8%)	173 (74.2%)	38 (16.3%)	195 (83.7%)
Medium-sized city (50,000–150,000 inhabitants)	71 (19.3%)	12 (16.9%)	59 (83.1%)	10 (14.1%)	61 (85.9%)
Small town (<50,000 inhabitants)	42 (11.4%)	8 (19.0%)	34 (81.0%)	6 (14.3%)	36 (85.7%)
Rural environment	22 (6.0%)	4 (18.2%)	18 (81.8%)	4 (18.2%)	18 (81.8%)
Works as diabetes consultant					
Yes	31 (8.4%)	8 (25.8%)	23 (74.2%)	7 (22.6%)	24 (77.4%)
No	337 (91.6%)	76 (22.6%)	261 (77.4%)	51 (15.1%)	286 (84.9%)
Working experience (years)	368 (100%)	13.4 ± 9.8, 11.0 [14.8] *	14.1 ± 9.9, 12.0 [13.0] *	15.5 ± 10.5, 13.0 [17.3] *	13.6 ± 9.7, 12.0 [14.0] *

* Mean ± standard deviation, median [interquartile range].

**Table 2:** Statistically significant associations between DHTL level and community pharmacists’ awareness, attitudes, and experiences with digital health technologies (*p* < 0.05).

Characteristic		N = 368 (%)	Low DHTL (Score ≤ 32.5, n = 132)	Medium DHTL (32.5 < Score < 35.5, n = 72)	High DHTL (Score ≥ 35.5, n = 164)	*p* Value
Are you aware of the existence of mobile applications to support a healthy lifestyle?	Yes	84 (22.8%)	22 (26.2%)	13 (15.5%)	49 (58.3%)	0.02
	No	284 (77.2%)	110 (38.7%)	59 (20.8%)	115 (40.5%)	
How did you become aware of the existence of mobile applications that can benefit patients with diabetes? (Mobile applications to support a healthy lifestyle)?	I am not aware	284 (77.2%)	110 (38.7%)	59 (40.5%)	115 (20.8%)	0.01
	Internet search	49 (13.3%)	10 (20.4%)	6 (12.2%)	33 (67.4%)	
	Conversation with colleagues and other healthcare professionals	18 (4.9%)	5 (27.8%)	4 (22.2%)	9 (50.0%)	
	Talking to patients	3 (0.8%)	2 (66.7%)	1 (33.3%)	0 (0.0%)	
	Pharmaceutical companies, Wholesalers of medicines and medical devices	9 (2.4%)	5 (55.6%)	0 (0.0%)	4 (44.4%)	
	Professional and scientific magazines and other literature	5 (1.4%)	0 (0.0%)	2 (40.0%)	3 (60.0%)	
Can knowledge of mobile applications that can be used by patients with diabetes help you in working with these patients?	Yes, almost always	55 (14.9%)	13 (23.6%)	8 (14.6%)	34 (61.8%)	<0.001
	Yes, often	160 (43.5%)	51 (31.9%)	34 (21.2%)	75 (46.9%)	
	Partially	108 (29.3%)	43 (39.8%)	23 (21.3%)	42 (38.9%)	
	Rarely	27 (7.3%)	14 (51.8%)	5 (18.5%)	8 (29.7%)	
	No, not at all	18 (4.9%)	11 (61.1%)	2 (11.1%)	5 (27.8%)	
Which way of conducting education about digital technologies, including mobile applications that can be used by patients with diabetes, would be most useful to you?	Face-to-face education	155 (42.1%)	77 (49.7%)	25 (16.1%)	53 (34.2%)	<0.001
	Online education in real time	71 (19.3%)	21 (29.6%)	14 (19.7%)	36 (50.7%)	
	Online education for self-study	142 (38.6%)	34 (23.9%)	33 (23.2%)	75 (52.9%)	
How would you like to receive current information about existing and new digital technologies, including mobile applications, that can be used by patients with diabetes?	In person, via e-mail address	192 (52.2%)	55 (28.6%)	43 (22.4%)	94 (49.0%)	0.03
	Through professional publications	20 (5.4%)	7 (35.0%)	5 (25.0%)	8 (40.0%)	
	Continuing Professional Development (CPD) courses	156 (42.4%)	70 (44.9%)	24 (15.4%)	62 (39.7%)	

Note: Table 2 presents variables that showed statistically significant associations (*p* < 0.05) related to community pharmacists’ awareness, attitudes, and experiences with digital health technologies. Non-significant findings are available in Supplementary Materials Table S1; DHTL—Digital Health Technology Literacy.

**Table 3:** Categories of Mobile Applications and the Number (N) and Percentage (%) of Community Pharmacists Aware of Their Use.

Mobile Application Category	Healthy Lifestyle (N = 84, 22.8%)	Diabetes Self-Management and Therapy Management (N = 58, 15.8%)
General Health and Wellness Tracking	35 (41.67%)	4 (6.90%)
Physical Activity Tracking	14 (16.67%)	—
Weight Management, Nutrition, and Diet Tracking	11 (13.10%)	—
Diabetes Management	8 (9.52%)	9 (15.52%)
Blood Glucose Monitoring	—	23 (39.66%)
Multi-Functional Medical Software	—	2 (3.45%)
Medication and Drug Information	—	1 (1.72%)
Health Insurance Management	1 (1.19%)	—
Other	24 (28.57%)	24 (41.38%)

**Table 4:** Patient Support Questions Reported by Community Pharmacists: Users vs. Non-Users of Diabetes-Related Mobile Applications.

Category	Questions Reported by Pharmacists from App-Using Patients (N = 18, 4.9%)		Questions Reported by Pharmacists from Non-App-Using Patients (N = 19, 5.2%)
Most Frequently Asked Questions		Most Frequently Asked Questions	
General clarifications about use of the application	4 (22.2%)	Which application (evidence-based) should I install?	3 (15.8%)
How to set up the application and log in?	1 (5.6%)	How do diabetes management applications work?	1 (5.3%)
Can I use the application multiple times a day?	1 (5.6%)	Which application provides the simplest visual presentation of glucose levels?	1 (5.3%)
How to record blood glucose level and frequency?	1 (5.6%)	Which application is the most reliable?	1 (5.3%)
Not Specified	11 (61.1%)	Which mobile applications can help me?	1 (5.3%)
		How are applications connected with glucose meters?	1 (5.3%)
		Are there applications for diabetes management?	1 (5.3%)
		What is the name of an application?	1 (5.3%)
		Not Specified	9 (47.4%)
Type of Diabetes of Patients Seeking Support		Type of Diabetes of Patients Seeking Support	
Type 1	6 (33.3%)	Type 1	4 (21.1%)
Type 2	2 (11.1%)	Type 2	3 (15.8%)
Both Types	10 (55.6%)	Both Types	12 (63.2%)
Age of Patients Seeking Support		Age of Patients Seeking Support	
≤30 years	7 (38.9%)	≤30 years	7 (36.8%)
31–65 years	11 (61.1%)	31–65 years	11 (57.9%)
65+ years	0 (0.0%)	65+ years	2 (10.5%)

Note: Percentages are calculated based on the number of pharmacists reporting questions in each subgroup. Age groups reflect pharmacists’ general impressions from routine interactions, not patient records, and no identifiable patient data were collected. Questions represent patient-initiated inquiries as reported by pharmacists.

**Table 5:** Characteristics of Training Received by Community Pharmacists on Mobile Applications for Diabetes Self-Management and Other Digital Technologies.

Category	Training on Mobile Applications (N = 9, 2.4%)		Training on Other Digital Technologies (N = 43, 11.7%)
Category of Technology for Which Pharmacists Received Training		Category of Technology for Which Pharmacists Received Training	
Blood Glucose Monitoring	55.6%	Health Monitoring and Diagnostics	18.4%
General Diabetes Management	11.1%	eHealth and Digital Records	27.9%
Not specified	33.3%	Pharmacy and Medical Software	27.9%
		Data Analysis and Research	4.6%
		General Computer Skills	2.3%
		Not specified	25.6%
Type of Training Received		Type of Training Received	
Continuing Professional Development (CPD) courses/Workshop	66.7%	Continuing Professional Development (CPD) courses/Workshop	67.4%
Symposium/Congress	22.2%	Symposium/Congress	11.6%
Do not remember	11.1%	Do not remember	20.9%
		Other	11.6%
Training Organizer		Training Organizer	
Health institution	22.2%	Health institution	37.2%
Pharmaceutical company	36.4%	Pharmaceutical company	23.3%
Wholesalers of medicines and medical devices	33.3%	Wholesalers of medicines and medical devices	4.7%
The Pharmaceutical Chamber of Serbia	11.1%	The Pharmaceutical Chamber of Serbia	20.9%
Pharmacists’ associations	11.1%	Other organizations	2.3%
Health institution	22.2%	Do not remember	34.9%

Note: Participants could specify more than one mobile application/digital technology and select more than one option for both the type of training and the training organizer.

## Data Availability

The original contributions presented in this study are included in the article/Supplementary Material. Further inquiries can be directed to the corresponding author.

## Supplementary Materials

The following supporting information can be downloaded at: https://www.mdpi.com/article/10.3390/pharmacy14020039/s1, Table S1: Non-Significant Associations Between DHTL Level and Community Pharmacists’ Awareness, Attitudes, and Experiences with Digital Health Technologies (*p* ≥ 0.05); File S2: Questionnaire Used in the Study.
