# Supplementary material for: Exploring Community Pharmacists’ Awareness, Attitudes, and Experiences with Digital Health Technologies: A Focus on Mobile Applications for Diabetes Mellitus Self-Management
Source: Pharmacy (Basel). 2026 Mar 2;14(2):39. doi: 10.3390/pharmacy14020039 (PMC13010657; doi:10.3390/pharmacy14020039)
Supplement: Supplementary file 1 [file pharmacy-14-00039-s001.zip › pharmacy-4125657-supplementary.pdf]

Table S1. Non-Significant Associations Between DHTL Level and Community Pharmacists' Awareness, Attitudes, and Experiences with Digital Health Technologies (p ≥ 0.05)

Characteristic		N = 368 (%)	Low DHTL (score <= 32.5, n = 132)	Medium DHTL (32.5 > score < 35.5, n = 72)	High DHTL (score => 35.5, n = 164)	p value
Are you aware of mobile applications to support disease self-management and therapy management?	Yes	58 (15.8%)	16 (27.6%)	10 (17.2%)	32 (55.2%)	0.2
	No	310 (84.2%)	116 (37.4%)	62 (20.0%)	132 (42.6%)	
How often do you recommend patients with diabetes to use the Mobile Application to support a healthy lifestyle?	I am not aware	284 (77.2%)	110 (38.7%)	59 (20.8%)	115 (40.5%)	0.51
	Never or very rarely (once a year or less)	39 (10.6%)	11 (28.2%)	7 (18.0%)	21 (53.8%)	
	Rarely (less than once a month)	14 (3.8%)	3 (21.4%)	2 (14.3%)	9 (64.3%)	
	Occasionally (several times a month)	23 (6.3%)	6 (26.1%)	4 (17.4%)	13 (56.5%)	
	Often (several times a week)	4 (1.1%)	1 (25.0%)	0 (0.0%)	3 (75.0%)	
	Always (everyday)	4 (1.1%)	1 (25.0%)	0 (0.0%)	3 (75.0%)	
How often do you recommend diabetes patients to use the Mobile Application to support disease	I am not aware	310 (84.2%)	116 (37.4%)	62 (20.0%)	132 (42.6%)	0.5
	Never or very rarely (once a year or less)	27 (7.3%)	9 (33.3%)	5 (18.5%)	13 (48.2%)	

self-management and therapy management?	Rarely (less than once a month)	10 (2.7%)	2 (20.0%)	2 (20.0%)	6 (60.0%)	
	Occasionally (several times a month)	12 (3.3%)	4 (33.3%)	3 (25.0%)	5 (41.7%)	
	Often (several times a week)	6 (1.6%)	0 (0.0%)	0 (0.0%)	6 (100.0%)	
	Always (everyday)	3 (0.8%)	0 (0.0%)	0 (0.0%)	2 (100.0%)	
How did you become aware of the existence of mobile applications that can benefit patients with diabetes? (Mobile applications to support disease self-management and therapy management)?	I am not aware	310 (84.2%)	116 (37.4%)	62 (20.0%)	132 (42.6%)	0.42
	Internet search	18 (4.9%)	5 (27.8%)	2 (11.1%)	11 (61.1%)	
	Conversation with colleagues and other healthcare professionals	14 (3.8%)	4 (28.6%)	2 (14.3%)	8 (57.1%)	
	Talking to patients	3 (0.8%)	1 (33.3%)	2 (66.7%)	0 (0.0%)	
	Pharmaceutical companies, Wholesalers of medicines and medical devices	20 (5.4%)	6 (30.0%)	3 (15.0%)	11 (55.0%)	
	Professional and scientific magazines and other literature	3 (0.8%)	0 (0.0%)	1 (33.3%)	2 (66.7%)	
In your opinion, are patients with diabetes interested	Yes, they are almost always interested	9 (2.4%)	4 (44.5%)	2 (22.2%)	3 (33.3%)	0.34

in using mobile applications in disease control?	Yes, they are often interested	27 (7.3%)	9 (33.3%)	3 (11.1%)	15 (55.6%)	
	They are partially interested	160 (43.5%)	48 (30.0%)	34 (21.3%)	78 (48.7%)	
	They are rarely interested	133 (36.1%)	52 (39.1%)	25 (18.8%)	56 (42.1%)	
	They are completely disinterested	39 (10.6%)	19 (48.7%)	8 (20.5%)	12 (30.8%)	
Indicate which categories of patients with diabetes, in your opinion, are interested in using mobile applications in disease control?	Mostly patients with type 1 diabetes	87 (26.4%)*	29 (33.3%)	13 (15.0%)	45 (51.7%)	0.46
	Mostly patients with type 2 diabetes	64 (19.5%)*	25 (39.1%)	15 (23.4%)	24 (37.5%)	
	All patients with diabetes	178 (54.1%)*	59 (33.2%)	36 (22.2%)	83 (46.6%)	
If the patient is already using a mobile application for better disease control, do they turn to you for help or advice regarding the use of the given mobile application?	Yes	18 (4.9%)	6 (33.3%)	2 (11.1%)	10 (55.6%)	0.6
	No	350 (95.1%)	126 (36.0%)	70 (20.0%)	154 (44.0%)	
If the patient does not use the mobile application for better disease control, do they turn to you for the	Yes	19 (5.2%)	7 (36.9%)	2 (10.5%)	10 (52.6%)	0.62

recommendation of the mobile application and/or help in connection with the use of the mobile application?	No	349 (94.8%)	125 (35.8%)	70 (20.1%)	154 (44.1%)	
Have you had any kind of training on mobile applications that can be used by diabetic patients?	Yes	9 (2.4%)	3 (33.3%)	2 (22.2%)	4 (44.5%)	1,000
	No	359 (97.6%)	129 (35.9%)	70 (19.5%)	160 (44.6%)	
Have you had any other type of training in the use of digital technologies in the provision of pharmaceutical health care? - electronic tools, systems, devices and resources that generate, store or process data.	Yes	43 (11.7%)	11 (25.6%)	9 (20.9%)	23 (53.5%)	0.31
	No	325 (88.3%)	121 (37.2%)	63 (19.4%)	141 (43.4%)	
In your opinion, should pharmacists provide advice and train patients for the adequate use of mobile applications to support a healthy lifestyle?	Yes	260 (70.7%)	85 (32.7%)	50 (19.2%)	125 (48.1%)	0.08
	No	108 (29.3%)	47 (43.51%)	22 (20.4%)	39 (36.1%)	

In your opinion, should pharmacists provide advice and train patients on the adequate use of mobile applications to support disease self-management and therapy management?	Yes	288 (78.3%)	97 (33.7%)	58 (20.1%)	133 (46.2%)	0.25
	No	80 (21.7%)	35 (43.8%)	14 (17.5%)	31 (38.7%)	
In your opinion, is it necessary to conduct additional training of pharmacists regarding the use of mobile applications, such as digital technologies that can be used in the provision of pharmaceutical health care?	Yes	322 (87.5%)	112 (34.8%)	65 (20.2%)	145 (45.0%)	0.48
	No	46 (12.5%)	20 (43.5%)	7 (15.2%)	19 (41.3%)	
In your opinion, is it necessary to conduct additional training of pharmacists regarding the use of mobile applications, such as applications that can be used by patients with diabetes?	Yes	330 (89.7%)	116 (35.2%)	67 (20.3%)	147 (44.5%)	0.51
	No	38 (10.3%)	16 (42.1%)	5 (13.2%)	17 (44.7%)	
Who, in your opinion, should conduct the	The institution where you are employed	64 (17.4%)	25 (40.6%)	13 (20.3%)	26 (40.6%)	0.39

education of pharmacists regarding the use of mobile applications, such as applications that can be used by patients with diabetes?	Pharmaceutical companies	77 (20.9%)	26 (33.8%)	11 (14.3%)	40 (51.9%)	
	Wholesalers of medicines and medical devices	11 (3.0%)	1 (9.1%)	2 (18.2%)	8 (72.7%)	
	Mobile App / Digital Technology Owner	69 (18.8%)	24 (34.8%)	16 (23.2%)	29 (42.0%)	
	Pharmaceutical Chamber	129 (35.1%)	46 (35.7%)	27 (20.9%)	56 (43.4%)	
	Faculty of Pharmacy	18 (4.9%)	10 (55.5%)	3 (16.7%)	5 (27.8%)	
Who, in your opinion, should conduct the education of pharmacists regarding the use of digital technologies that they can use in the provision of pharmaceutical health care?	The institution where you are employed	80 (21.7%)	36 (45.0%)	13 (16.3%)	31 (38.7%)	0.27
	Pharmaceutical companies	64 (17.4%)	21 (32.8%)	12 (18.8%)	31 (48.4%)	
	Wholesalers of medicines and medical devices	8 (2.2%)	2 (25.0%)	0 (0.0%)	6 (75.0%)	
	Mobile App / Digital Technology Owner	58 (15.8%)	19 (32.8%)	17 (29.3%)	22 (37.9%)	
	Pharmaceutical Chamber	133 (36.1%)	43 (32.3%)	25 (18.8%)	65 (48.9%)	
	Faculty of Pharmacy	25 (6.8%)	11 (44.0%)	5 (20.0%)	9 (36.0%)	
Would it be beneficial to your work with patients with diabetes if there were official	Yes	339 (92.1%)	118 (34.8%)	68 (20.1%)	153 (45.1%)	0.38

guidelines on mobile applications for their use?	No	29 (7.9%)	14 (48.3%)	4 (13.8%)	11 (37.9%)

*N=329 (those who believe that patients with diabetes are interested in using mobile applications for self-management of the disease); DHTL - Digital Health Technology Literacy

File S2. **Questionnaire Used in the Study**

A. **Socio-Demographic data**

1. Gender
   a. Male
   b. Female

2. You practice pharmaceutical care within:
   a. Pharmacies within Pharmacy Chains (state property)
   b. Pharmacies within Pharmacy Chains (private)
   c. Independent pharmacies

3. By education you are:
   a. Master of Pharmacy
   b. Master of Pharmacy - Specialist
   c. Master of Pharmacy - PhD

4. If you marked the answer under b in question number 3 , please indicate which specialization you have completed?

5. How many years of experience do you have in pharmaceutical care? (enter the number of years)

6. The place where you work is:
   a. A large city with more than 150,000 inhabitants
   b. A medium-sized city with 50,000-150,000 inhabitants
   c. A small town, with less than 50,000 inhabitants
   d. Rural area

7. Do you work as a "Diabetes Advisor" in accordance with the project of the Serbian Chamber of Pharmacy to provide a standardized service to patients with diabetes?
   a. Yes

b. No

B. Digital Health Technology Literacy – Assessment Questionnaire - Serbian version

Use of digital health applications

8. Below are the various statements related to applications on smartphones, tablets, etc. Please read the statements provided and indicate whether you agree with the statements

	Statement	I do not agree	I have no experience	I agree
**1**	I can record my health information through the application			
**2**	I can use the health information recorded through the application for my health			
**3**	I can record the volume of physical activity (e.g. number of steps), body mass and meals via the application			
**4**	I can check the volume of physical activity (e.g. number of steps), body mass and meals recorded through the application			

Use of the Internet for Health

9. Below listed terms are related to the use of computers or smartphones and the Internet. Please mark the terms that are familiar to you.

□ *Play* store (application store)	□ *Bluetooth*	□ *Wearable* devices
□ Web browser	□ Application (*App*)	□ Voice assistant (*Chatbot*)
□ *Cloud*	□ Domain (URL)	□ Update (synchronization)
□ *QR* code	□ Search Bar	□ I don¢t know any of the terms

10. Below are the various statements related to applications on smartphones, tablets, etc. Please read the statements provided and indicate whether you agree with the statements

	Statement	I do not agree	I have no experience	I agree
**1**	I can easily find an application that will help my health			

2	I can find more reliable applications by comparing different applications			

11. Listed statements are related to difficulties when using health information from the Internet and digital health technologies or services. Please read the listed statements and indicate whether you agree with the statements.

	Statement	I do not agree	I have no experience	I agree
1	I can judge whether information on the Internet or within digital health technologies is reliable			
2	I can judge whether information on the Internet or within digital health technologies is used for commercial purposes			
3	I can check if the same information is available on other websites or other sources on the Internet			
4	I can judge whether the information I find on the Internet or within digital health technologies is adequate to use			
5	I can use information I find on the Internet or within digital health technologies to make health-related decisions			

Applications setup

12. Below are the various statements related to applications on smartphones, tablets, etc. Please read the statements provided and indicate whether you agree with the statements.

	Statement	I do not agree	I have no experience	I agree
1	I can log in to use the application (create an account, password, etc.)			
2	I can update the application			
3	I can adjust the options in the application according to my wishes (sound, security, screen, notifications, etc.)			

C. Mobile applications that can be used by patients with diabetes

13. Please indicate how you get informed about news in the field of diabetes? (check all that apply)
    a. Internet search
    b. Conversation with colleagues and other healthcare professionals

c. Talking to patients
d. Continuing medical education
e. Pharmaceutical companies
f. Professional journals and other literature
g. I do not keep up to date with diabetes news

14. If you answered question number 22 under a, please indicate which websites you visit most often?

15. Are you aware of the existence of mobile applications that can be useful for patients with diabetes? (mark your answer for both categories of mobile applications)

	Yes	No
Mobile applications to support a healthy lifestyle		
Mobile applications to support disease self-management and therapy management		

16. **a.** If you answered question number 24 that you are aware of mobile applications to support a healthy lifestyle , please indicate the names of the mobile applications you are aware of:

b. If you answered question number 24 that you are aware of mobile applications for supporting disease self-control and therapy management , please indicate the names of the mobile applications you are aware of:

17. If you answered yes to question number 24 for any of the categories, how often do you recommend the use of those mobile applications to patients with diabetes? (check your answer for the category/s of mobile apps you are aware of)

	The answer to question 24 is I am not aware of it	Never or very rarely (once a year or less)	Rarely (less than once a month)	Occasionally (several times a month)	Often (several times a week)	Always (everyday)

Mobile applications to support a healthy lifestyle						
Mobile applications to support disease self-management and therapy management						

18. If you answered yes to question number 24 for any of the categories, please indicate how you became aware of the existence of mobile applications that can be useful for patients with diabetes? (check your answer for the category/s of mobile apps you are aware of)

	The answer to question 24 is I am not aware of it	Internet search	Conversation with colleagues and other healthcare professionals	Talking to patients	Pharmaceutical companies, Wholesalers of drugs and medical devices	Professional and scientific magazines and other literature
Mobile applications to support a healthy lifestyle						
Mobile applications to support disease self-management and therapy management						

19. In your opinion, are patients with diabetes interested in using mobile applications in disease control?

    a. Yes, they are almost always interested
    b. Yes, they are often interested.
    c. partially interested.
    d. They are rarely interested.

e. They are completely uninterested.

20. **a.** If you circled the answer from a to d to question number 28 , please indicate which categories of patients with diabetes (type of diabetes) are, in your opinion, interested in using mobile applications in disease control?

    a. Mostly patients with type 1 diabetes
    b. Mostly patients with type 2 diabetes
    c. All patients with diabetes

    b. If you circled the answer from a to d to question number 28 , please indicate which categories of patients with diabetes (age) are in your opinion interested in using mobile applications in disease control? (you can mark more than one answer)

    a. Patients of younger age (≤ 30 years)
    b. Middle-aged patients (31-65 years)
    c. Elderly patients (65+ years)

21. If the patient **already uses** a mobile application **for better disease control** , does he turn to you for help or advice regarding the use of the given mobile application?

    a. Yes
    b. No

22. If you answered yes to question number 30, please indicate which applications are in question:

23. If you answered yes to question number 30, please list the most common questions you receive from diabetes patients about the use of mobile applications they already use?

24. **a.** If you answered yes to question number 30 , please indicate from which category of patients with diabetes (Type of diabetes) you most often receive questions **about the use of mobile applications** ?

    c. Mostly from patients with type 1 diabetes
    d. Mostly from patients with type 2 diabetes
    e. From all patients regardless of the type of diabetes

b. If you answered yes to question number 30 , please indicate from which category of patients with diabetes (Age of patients) you most often receive questions **about the use of mobile applications** ? (check all that apply)

a. Patients of younger age (≤ 30 years)
b. Middle-aged patients (31-65 years)
c. Elderly patients (65+ years)

25. If the patient **does not use** the mobile application for better disease control , do they turn to you for recommendations and help regarding the use of the mobile application?
    a. Yes
    b. No

26. If you answered yes to question number 34, please list the most common questions you receive from diabetes patients for referrals and help regarding the need for mobile apps?

27. **a.** If you answered yes to question number 34, please indicate from which categories of patients with diabetes (Type of diabetes) do you most often receive questions for recommendations and help regarding the need for mobile applications?

    a. Mostly from patients with type 1 diabetes
    b. Mostly from patients with type 2 diabetes
    c. From all patients regardless of the type of diabetes

    b. If you answered yes to question number 34, please indicate from which categories of patients with diabetes (Age) you most often receive questions for recommendation and help regarding the need for mobile applications? (you can mark more than one answer)

    a. Patients of younger age (≤ 30 years)
    b. Middle-aged patients (31-65 years)
    c. Elderly patients (65+ years)

28. Have you had any kind of training on mobile applications that can be used by diabetic patients?
    a. Yes
    b. No

29. **a.** If you answered yes to question number 37, please indicate for which mobile applications the training was organized - the name of the application

    b. If you answered yes to question number 37, please indicate the type of mobile application training that was organized? (check all types of training you have had)

    a. Continuing Professional Development (CPD) course
    b. Symposium
    c. Congress
    d. Workshop
    e. Others
    f. I don't know (I don't remember)

    c. If you marked "Other" in the previous question , please specify other types of education

    d. If you answered yes to question number 37, please indicate who was the organizer of the training on mobile applications? (check all types of organizations that apply)

    a. Health care institution

    b. Pharmaceutical company

    c. Wholesalers of medicines and medical devices

    d. Pharmaceutical Chamber

    e Higher education institution

    f. Other organizations

    g. I don't know (I don't remember)

    e. If you marked "Other" in the previous question , please specify other organizations

30. Have you had any other type of training in the use of digital technologies in the provision of pharmaceutical care? By digital technology we mean electronic tools, systems, devices and resources that generate, store or process data.

    a. Yes
    b. No

31. **a.** If you answered yes to question number 39, please indicate for which digital technologies the training was organized - the name of the application?

b . If you answered yes to question number 39, please indicate the type of training on digital technologies that was organized? (check all types of training you have had)

a. Continuing Professional Development (CPD) course
b. Symposium
c. Congress
d. Workshop
e. Others
f. I don't know (I don't remember)

c. If you marked "Other" in the previous question , please specify other types of education

d. If you answered yes to question number 39, please indicate who was the organizer of the training on digital technologies? (check all types of organizations that apply)

a. Health care institution

b. Pharmaceutical company

c. Wholesalers of medicines and medical devices

d. Pharmaceutical Chamber

e Higher education institution

f. Other organizations

g. I don't know (I don't remember)

e. If you marked "Other" in the previous question , please specify other organizations

32. Can knowledge of mobile applications that can be used by patients with diabetes help you in working with these patients?
    a. Yes, almost always.
    b. Yes, often.
    c. Partially
    d. Rarely
    e. No, not at all.

33. In your opinion, should pharmacists provide advice and train patients for adequate use of mobile applications? (mark your answer for both categories of mobile applications)

	Yes	No
Mobile applications to support a healthy lifestyle		
Mobile applications to support disease self-management and therapy management		

34. In your opinion, is it necessary to conduct additional training of pharmacists regarding the use of mobile applications and digital technologies? (check your answer for both categories)

	Mobile applications, such as applications that can be used by patients with diabetes	Digital technologies* that can be used in the provision of pharmaceutical care
Yes		
No		

*By digital technology we mean electronic tools, systems, devices and resources that generate, store or process data.

35. Who, in your opinion, should conduct the education of pharmacists regarding the use of mobile applications and digital technologies? (check your answer for both categories)

	The institution where you are employed	Pharmaceutical companies	Wholesalers of medicines and medical devices	Mobile application owner	Pharmaceutical Chamber	Faculty of Pharmacy
Mobile applications, such as applications that can be used by patients with diabetes						

Digital technologies* that can be used in the provision of pharmaceutical care							

*By digital technology we mean electronic tools, systems, devices and resources that generate, store or process data.

36. What method of delivering education on digital technologies — including mobile applications that can be used by patients with diabetes — would be most useful to you?

    a. Face-to-face education
    b. Online education in real time
    c. Online education for self-study

37. How would you like to receive current information about existing and new digital technologies including mobile applications that can be used by patients with diabetes?

    a. In person, via email address
    b. Through professional publications
    c. Continuing medical education

38. Would it be helpful for your work with patients with diabetes to have official recommendations regarding mobile applications that diabetic patients can use?

    a. Yes
    b. No
